# Characteristics of Suspected COVID-19 Discharged Emergency Department Patients Who Returned During the First Wave

**DOI:** 10.5811/westjem.58717

**Published:** 2023-04-03

**Authors:** Jonathan Gong, Rene Mayorga, Roland Hentz, Martin Lesser, Seleshi Demissie, Frederick Davis, Adam Berman, Matthew Barish, Stuart L. Cohen, Kate L. van Loveren, Nancy S. Kwon, Northwell COVID-19 Research Consortium

**Affiliations:** * Zucker School of Medicine at Hofstra/Northwell, Long Island Jewish Medical Center, Department of Emergency Medicine, New Hyde Park, New York; † Northwell Health, Emergency Medicine Service Line, New Hyde Park, New York; ‡ Feinstein Institute for Medical Research, Biostatistics Unit, Great Neck, New York; § Zucker School of Medicine at Hofstra/Northwell, Department of Molecular Medicine & Department of Population Health, Hempstead, New York; || Donald & Barbara Zucker School of Medicine at Hofstra/Northwell, Northwell Health, Department of Radiology, Hempstead, New York

## Abstract

**Introduction:**

Limited information exists on patients with suspected coronavirus disease 2019 (COVID-19) who return to the emergency department (ED) during the first wave. In this study we aimed to identify predictors of ED return within 72 hours for patients with suspected COVID-19.

**Methods:**

Incorporating data from 14 EDs within an integrated healthcare network in the New York metropolitan region from March 2–April 27, 2020, we analyzed this data on predictors for a return ED visit—including demographics, comorbidities, vital signs, and laboratory results.

**Results:**

In total, 18,599 patients were included in the study. The median age was 46 years old [interquartile range 34–58]), 50.74% were female, and 49.26% were male. Overall, 532 (2.86%) returned to the ED within 72 hours, and 95.49% were admitted at the return visit. Of those tested for COVID-19, 59.24% (4704/7941) tested positive. Patients with chief complaints of “fever” or “flu” or a history of diabetes or renal disease were more likely to return at 72 hours. Risk of return increased with persistently abnormal temperature (odds ratio [OR] 2.43, 95% CI 1.8–3.2), respiratory rate (2.17, 95% CI 1.6–3.0), and chest radiograph (OR 2.54, 95% CI 2.0–3.2). Abnormally high neutrophil counts, low platelet counts, high bicarbonate values, and high aspartate aminotransferase levels were associated with a higher rate of return. Risk of return decreased when discharged on antibiotics (OR 0.12, 95% CI 0.0–0.3) or corticosteroids (OR 0.12, 95% CI 0.0–0.9).

**Conclusion:**

The low overall return rate of patients during the first COVID-19 wave indicates that physicians’ clinical decision-making successfully identified those acceptable for discharge.

## INTRODUCTION

The coronavirus disease 2019 (COVID-19) outbreak was declared a pandemic by the World Health Organization on March 11, 2020.^1^ At that time, emergency departments (ED) and hospitals in the United States, and specifically New York City, became inundated with patients with respiratory concerns for a disease with evolving diagnostics and therapeutics. The COVID-19 outbreak quickly spread throughout New York State at an unprecedented rate with the peak of hospitals’ capacity occurring on April 9, 2020.^2^–^4^ Many patients presenting to EDs were evaluated, and their disposition was made largely without confirmatory testing. Thus far, little is known about the subsequent healthcare encounters of patients who were discharged from the ED with COVID-19 or suspected COVID-19 and the factors that may have increased their risk for return. We hope to better understand the role of EDs during this outbreak and the outcomes of treat-and-release patients with suspected COVID-19.

Although recent studies have looked at clinical characteristics and risk factors for poor outcomes in hospitalized patients with COVID-19, sparse data exists for the ED setting.^5^–^12^ During the initial surge of COVID-19 in New York, there were no evidence-based guidelines to help clinicians care for patients in the ED. The role of the ED in evaluating patients with suspected COVID-19 and determining disposition was instrumental during this ongoing public health crisis. Limited inpatient beds and overall resources, such as COVID-19 testing and mechanical ventilators, forced emergency clinicians to use surrogate markers of critical illness—vital signs, laboratory data, and radiologic data—to determine whether patients with suspected COVID-19 required admission. Further, during the first COVID-19 surge there were dynamic changes in clinical decision-making and admission criteria. As our knowledge of COVID-19 evolved and resources remained limited, data on outcomes of patients who were discharged from the ED with suspected COVID-19 would assist in the development of future clinical guidelines.

Our main study objective was to understand how ED care was delivered during the first wave of the novel COVID-19 pandemic when resources and therapeutics were severely limited. Specifically, we aimed to achieve this objective by characterizing the demographics, baseline comorbidities, presenting clinical tests, and outcomes of patients with suspected COVID-19 who were discharged from an academic healthcare system at the epicenter of the pandemic. Understanding this information is vital in identifying patients who were at the highest risk of returning to the ED potentially due to worsening COVID-19 infection. Findings from this study may also highlight those patients who were safely discharged from the ED despite the acuity of their presenting complaint. This investigation can assist healthcare systems in developing future disaster protocols and allocating limited resources.

## MATERIALS AND METHODS

### Study Design and Setting

This was a retrospective chart review of consecutive patients with suspected COVID-19 who were seen in one of 14 EDs at 13 hospitals within an integrated healthcare network. This health system serves approximately 11 million persons in the New York City metropolitan area. The study was performed with institutional review board approval and waiver of informed consent. As a general quality metric, EDs track patients who return for a second visit to the ED within 72 hours to assess possible misdiagnosis or treatment failures. Due to the availability of this metric in our health system and in other health systems, we specifically assessed the characteristics of patients with suspected COVID-19 who returned to the ED within 72 hours of discharge and who required hospital admission on their second visit. Patients with suspected COVID-19 were included mainly because testing and delays in results were extremely limited during this period with turnaround times of 72–96 hours. Thus, patients’ COVID-19 status was not known at the time of disposition.

**Population Health Research Capsule**

What do we already know about this issue?

*There is limited data about discharged patients from the emergency department (ED) and those at risk for requiring further care due to progression of disease*.

What was the research question?

*We sought to identify factors that increased the risk of coronavirus disease 2019 (COVID-19) patients returning to the hospital within 72 hours*.

What was the major finding of the study?

*2.9% returned within 72 hours and 95% were then admitted. Patients with increased age (OR 1.37 per decade), abnormal temperature (OR 2.43), and abnormal chest radiograph (OR 2.54) are at higher odds of returning to the ED*.

How does this improve population health?

*Our findings can help emergency physicians and outpatient clinicians caring for suspected COVID-19 patients, as most can be treated as an outpatient*.

Patients were included if they had an initial ED visit between March 2–April 27, 2020. This timeframe represents a bell curve of cases that presented to the ED during the initial surge of the COVID-19 pandemic in the New York City metropolitan area. The EDs were classified based on their available services and included one freestanding ED, six community EDs, six tertiary EDs, and one tertiary pediatric ED. We selected these 14 EDs because they use the same electronic health record (EHR) (Sunrise Emergency Care, Allscripts Healthcare Solutions, Inc., Chicago, IL).

#### Selection of Participants

We collected data on consecutive adult patients ≥18 years with suspected COVID-19 who were discharged from the ED. To determine suspicion of COVID-19, participants needed to meet two criteria. The first criteria for inclusion was presentation to the ED with a chief complaint related to “viral illness” (See Table 1). The second criteria for inclusion was documentation of either a COVID-19-related discharge diagnosis or discharge instructions containing verbiage such as “quarantine” or “stay at home” that started in March 2020 at the beginning of the pandemic (See Table 1 and Appendix A). For example, a patient could meet the first criteria for inclusion with an initial chief complaint of “flu.” To meet the second criteria for inclusion, the patient would need either a COVID-19-related discharge diagnosis or a set of discharge instructions containing phrases such as “quarantine,” “14-day,” or “stay at home.”

Patients could have an ICD-10 discharge diagnosis of “pain” or “headache” that was not COVID-19 related, but their discharge instructions contained pandemic-related verbiage, which meets the second criteria for inclusion. Or, they could have a COVID-19 related discharge diagnosis of “COVID-19” and have discharge instructions that did not contain pandemic-related verbiage. Discharge from the ED was defined as having an ED disposition of “discharged” or “left against medical advice.” Patients were excluded if they were registered in the ED and left without being seen by a clinician or were admitted or transferred on their first visit. Included patients were divided into two cohorts: 1) patients who did not return within 72 hours; and 2) patients who returned within 72 hours.

#### Measurements and Outcomes

We collected initial triage vital signs and discharge vital signs. Vital signs were classified as normal–yes or no–based upon clinical relevance (See Appendix B). To account for the possibility of improvement or worsening of vital signs after administration of therapies, multiple categories were identified for each patient: normal to normal; normal to abnormal; abnormal to normal; and abnormal to abnormal. Laboratory test variables from a patient’s first visit were classified either as a normal value, an abnormal high value, or an abnormal low value depending on clinical significance. Laboratory testing was based upon the treating clinician’s discretion, and well-presenting patients may not have required laboratory values. If a laboratory test was not ordered at the discretion of the clinician, it was classified as “not ordered.” These “not ordered” laboratory values had no impact on the decision to discharge the patient as the values were not available to the clinicians at the time of disposition decision. These values were assumed to be normal had they had been officially ordered, and thus were classified as such.

Findings from chest radiograph (CXR) reports from the initial ED visit were extracted and then analyzed using a natural language processing computer model developed by our institution’s radiology department. The model was built in a stepwise iterative method. An initial model was designed by asking radiologists for common terms used to describe common lung pathology or the absence of lung pathology and review of 100 cases by manual annotation of key terms and phrases. This served as the initial model. Three random samples of 100 studies were annotated by two radiologists (MAB, SLC) in a binary fashion (pathology/no pathology). The same annotated reports were then analyzed by the model with discrepancies analyzed by MAB and SLC.

The model was then manipulated after each of the three rounds of 100 studies to account for these discrepancies and additional phrases. This process (annotation followed by model testing) was then repeated until a threshold accuracy rate of >94% was achieved on all three samples of 100 cases within the iteration. Once the threshold was reached, the model was considered complete, and model statistics were tested on a random sample of 10 sets of 100 cases. In this set of 1,000 annotated cases, the model had an accuracy rate of 96.6%.

#### Analysis

We divided the included patients into two cohorts: patients discharged with no return within 72 hours; and patients discharged with a return visit within 72 hours. Predictor variables from each patient’s first visit were used to determine the predictors of a return to the ED within 72 hours.

Continuous variables are described by mean and standard deviation or median and interquartile range (IQR). Frequency counts and percentages are reported for categorical variables. Predictor variable importance was initially determined by testing the univariable associations between each variable and return to the ED within 72 hours. Age was tested using logistic regression, and categorical variables were tested using chi-square and Fisher exact tests. Predictor variables were selected for relevance if the *P*-value from this initial test was *P* < 0.1.

Next, predictors meeting this threshold were entered into a multivariable logistic regression model that was refined using backward elimination. Backward elimination continued until tests of association between each predictor variable in the model and return to the ED within 72 hours had *P*-values of *P* < 0.05. We performed logistic regression models using the identified important predictor variables to evaluate their association with return to the ED within 72 hours status. We classified laboratory variables into normal or missing, abnormal high, or abnormal low depending on clinical significance. All statistical analyses were performed using SAS software version 9.4 (SAS Institute Inc, Cary, NC).

## RESULTS

### Characteristics of Study Subjects

A total of 81,321 patients were seen in the ED during the study period, of whom 27,144 were identified as having a chief complaint of “viral illness” and 18,599 were identified as having “suspected COVID-19” as per our inclusion definitions. A total of 18,599 patients met the inclusion criteria.

The demographic distribution of included patients is shown in Table 2 and 3. The median age of patients who met inclusion criteria was 46 years (IQR 34–58, range 18–104). The gender distribution was 50.74% female (9,437), and 49.26% male (9,162). Most patients identified as not Hispanic or Latino (62.78%; 11,677) and the remaining were Hispanic or Latino (28.95%; 5,384) or of unknown ethnicity (8.27%; 1,538). With regard to race, 34.22% (6,365) of patients were White, 33.47% (6,226) identified as other/multiracial, 17.67% (3,287) were Black, 8.06% (1,499) were Asian, and 6.57% (1,222) were Native American/Alaskan/Hawaiian/Pacific Islander/unknown. Most patients were insured through commercial insurance including private insurance, insurance through an employer, or managed care (75.18%, 13,983).

**Figure 1:**
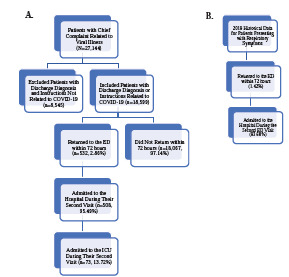
**A**. Eligibility criteria for study sample (N=18,599). **B**. Historical data on emergency department 72-hour return and admission from 2019 for patients presenting with respiratory symptoms. *COVID-19*, coronavirus 2019; *ED*, emergency department.

Of the 18,599 patients identified, 532 (2.86%) returned to the ED within 72 hours from their initial visit (See Figure 1a). The admission rate was 95.49% (508/532) for those who returned within 72 hours. Of these patients who were admitted to the hospital on their second visit, 73 (13.72%) were admitted to the intensive care unit (ICU). Historical data from 2019 showed a 72-hour return rate for patients presenting with respiratory symptoms of 1.42% and an admission rate of 93.68% on the second ED visit (See Figure 1b).

Males had significantly higher odds of return within 72 hours (odds ratio [OR] 1.41, 95% CI 1.2–1.7) (Table 3). Older patients had greater odds of returning than younger patients (10-year increment: OR 1.37, 95% CI 1.3–1.5 for 72-hour return). With regard to chief complaints and past medical history, patients with chief complaints of “fever” or “flu” or with a history of diabetes or renal disease were more likely to return at 72 hours. Patients with persistently abnormal temperature (OR 2.43, 95% CI 1.8–3.2) and respiratory rate (2.17, 95% CI 1.6–3.0) were more likely to return within 72 hours. Abnormal systolic blood pressure at triage or at discharge had lower odds of returning for evaluation. Of the laboratory tests, abnormally high neutrophil counts, abnormally elevated bicarbonate, abnormally low platelets, and abnormally elevated aspartate aminotransferase (AST) were associated with a higher rate of return within 72 hours (Table 3). Furthermore, patients with an abnormal CXR had higher odds of return admission (OR 2.54, 95% CI 2.0–3.2).

Among all patients, those discharged on antibiotics (2.77% of our study population) were significantly less likely to return at 72 hours (OR 0.12, 95% CI 0.0–0.3). Of the 516 patients who were discharged on antibiotics, five returned within 72 hours. Similarly, those discharged on corticosteroids (2.23% of the study population) were significantly less likely to return at 72 hours (OR 0.12, 95% CI 0.02–0.9). Of the 415 patients who received corticosteroids, only one patient (0.24%) returned within 72 hours.

## DISCUSSION

Despite the lack of evidence-based clinical guidelines in the ED setting during the initial surge in the New York City metropolitan area, our study found that the return rate was relatively low (<3%). While our sample looked at return rates within three days, two other studies by Husain et al and Berdahl et al noted return rates of 13.7% within 14 days and 24.7% within 30 days during this initial surge, respectively.^28^,^29^ Further, our admission rate for return patients presenting with respiratory concerns was lower when compared to the year prior. Considering the unprecedented circumstances and the novel presenting features of COVID-19, the admission rate was much lower than expected.

Our lower admission rates could be explained by the lack of availability of inpatient beds and the overall volume of critically ill patients, which necessitated discharge of patients who were stable. Disposition decisions for suspected COVID-19 patients were based largely on available vital signs, clinical gestalt, and laboratory results. Our findings further establish that despite not having definitive confirmation of COVID-19, emergency physicians can base their clinical decisions on results from more widely available resources to discharge patients, even with a novel disease. Moreover, our low return rate signifies that patients who were safe for discharge were reliably identified, which speaks volumes for potentially subsequent waves and future disasters.

Vital sign abnormalities in respiratory rate and temperature increased the odds of return to the ED in patients with suspected COVID-19. Of those within our analysis, persistent abnormal respiratory rate and temperature were noted to have the largest effect. While temperature is a concrete variable, respiratory rate can be subjective and at times undermeasured, it is important to highlight the effect on a patient of persistently abnormal respiratory rate, possibly as a sign of potential decompensation from this respiratory illness. We found that abnormalities in heart rates were not associated with increased odds of return, which differs from Husain et al and Margus et al.^28^,^29^ The Margus et al study differs in methodology from our paper and that of Husain et al in that the Margus study was a nested control trial, where patients were matched who returned within 72 hours. While bradycardia and tachycardia may clinically differ in a clinician’s decision-making, our analysis of the unadjusted rates of return was higher for patients with either bradycardia or tachycardia. This further supports grouping heart rate abnormalities together as abnormal.

Systolic blood pressure (BP) abnormalities decreased the likelihood of returning to the ED within 72 hours. Systolic BPs (SBP) that were not normal were defined as anything outside of the 90–140 range (Appendix B). Only 53 patients of this group were hypotensive during their ED stay, and only five of the 53 were noted to have returned within 72 hours. We were unable to separate the abnormal into high and low given the small number of hypotensive patients in this large sample, with hypotensive patients representing 0.28%. Thus, clinical judgment should be used when discharging patients who experienced hypotension during their ED stay. Patients presenting with hypertension, which was initially thought of as a predisposing factor based upon past medical history for worse outcomes, were noted to have a lower return rate, which also is contrary to Margus et al.

Diagnostic tests such as laboratory values and imaging can also impact the clinician’s disposition decision. Abnormal lung findings on a CXR increased the odds of return within 72 hours, similar to Margus et al. More subtle findings of an abnormally high AST, neutrophil counts, and bicarbonate level and abnormally low platelet counts were also indicative of a higher rate of return. This finding of transaminitis was also found to be a predictor for return in the sample from Husain et al.^28^ While each individual finding may have impact on the likelihood of return to the ED, the composite results of this study may lead toward prospective scoring tools that can better guide the clinician on disposition decisions for patients who present with COVID-19.

Among patients who did return to the ED within 72 hours, we found the subsequent admission rate to be almost 96%. In comparison to our data from 2019 for those patients presenting with respiratory symptoms, our system admitted a similar percentage (93.7%) of patients. More specifically, these patients who returned were noted to have significantly worsening symptoms, with approximately 13.7% requiring an ICU admission. This is consistent with the inpatient data published during the earlier surge with the finding that14.2% were treated in the ICU.^30^ Our study identifies factors that increases the odds for returning to the ED and admission to the hospital for patients with suspected COVID-19. The natural disease progression of COVID-19, like many other infectious respiratory illnesses, has the possibility of a patient requiring ICU admission and mechanical ventilation.

Other studies have also looked at clinical factors in the ED that could be predictive of worse outcomes or return to the ED. In our study, older age, abnormal temperature readings, increased respiratory rate, and abnormal CXRs predicted return in 72 hours. These results were similar to a recent study by Kilaru et al^12^ and to the discharge criteria used by Berdahl et al. In Kilaru’s study, only confirmed COVID-19 patients (1,419) were included, and their findings were similar with abnormal temperature, oxygen saturation, and CXR, and older age (≥60 years) having higher odds of return within 72 hours (66 patients) or 7 days (117).^12^ The findings in our study support some of the findings in these prior studies. Unlike the Kilaru study, we did not restrict our study population to patients with confirmed COVID-19. Instead, we included any patients under investigation for COVID-19 based upon chief complaints, discharge diagnoses, and discharge instructions related to COVID-19. During our study, only 7,941/18,599 patients (42.70%) were tested for COVID-19, of whom 4,704 (59.24%) tested positive (See Table 2). Testing for COVID-19 was a non-contributing factor in the disposition decision-making for the treating emergency physician, as these results were not available for 24–48 hours for the study period. Factors such as abnormal oxygen saturation, advanced age, abnormal temperature, and CXR results appear to be markers of COVID-19 disease severity.

Although the sample size is small, we must note the significantly decreased odds of returning within 72 hours for patients discharged on corticosteroids and antibiotics. During the study period, health system guidelines initially warned against the use of steroids, which was later changed upon identification of the inflammatory phase of the COVID-19 infection. These findings support early data on COVID-19-hospitalized patients requiring supplemental oxygen that showed corticosteroids could be beneficial. Currently, corticosteroids are a key therapy for hospitalized patients who require oxygen. More research is needed to evaluate the effect of corticosteroids for patients who are seen for COVID-19 in the ED or outpatient setting.^13^ Recent studies on the impact of antibiotics on COVID-19 have shown a lack of efficacy. However, in our small sample of patients who received antibiotics at discharge, the odds of return were also reduced. Given that our current sample is not fully composed of COVID-19 cases, administration of antibiotics and steroids could be effective in treating alternative diagnoses, like bacterial etiologies of infection.

Our study adds to the limited literature that describes patients who presented to the ED during the initial wave of COVID-19 in the New York City metropolitan area. Our study supports using clinically available data for clinicians to discharge suspected COVID-19 patients. However, given the nature of COVID-19 and the natural progression of the disease, strict return precautions must be provided to patients. Before these studies, risk factors were primarily extrapolated from in-patient studies, and based on experiences in China, Europe, and other countries that were affected by the pandemic before the US.^8^,^12^,^14^–^26^ Our study supports that many risk factors for severe disease found in hospitalized patients—older age and abnormal temperature, tachypnea, and CXR—were also present in discharged ED patients with suspected or confirmed COVID-19 who had higher odds of returning to the ED within 72 hours.^14^–^16^,^27^

## LIMITATIONS

While this retrospective cohort study allows us to identify associations between patient characteristics and return to the ED, it does not permit conclusions related to causality. This study was performed at a single health system in the Northeast, so our findings may not reflect national or international populations. They may have had a 72-hour return to an ED at different health system, death, or other morbidity that was not captured within the EHR shared by the 14 EDs from which we collected data. Further, neither do we know how mortality rates among those who returned within 72 hours compared to those who were initially admitted with COVID-19, information that could have provided deeper clinical insight into the effects of discharge.

Emergency departments track 72-hour returns as a quality measure and can be indicative for possible misdiagnosis and or treatment failures. Given the pathogenesis of COVID-19, 72-hour return may not encompass the progression of the COVID-19 disease process, although patients may present to the ED at different points during their disease. Their first visit could be on day 1 or 10 of symptoms; thus, allowing for a 72-hour return could allow for varying sequelae of COVID-19 depending on the day of their presentation. Husain et al and Berdhal et al extended the window of follow-up to 14 and 30 days, respectively; however, these follow-up windows may be more indicative of other pathology rather than the acute viral phase of COVID-19. Furthermore, we are not advocating that these patients require admission for that length of time.^30^,^31^ Within the 72-hour window, certain sequelae of COVID-19 may be missed, such as bacterial super-infections, deep vein thromboses, and neurologic complications that were seen as late complications of COVID-19.

Our cohort included patients based on presenting symptoms consistent with COVID-19. They did not always have a COVID-19 test to confirm the diagnosis, as the testing platform was limited at that time. Within our cohort that was tested, the positivity rate was 59.24%, although it must be noted that during the study period the accuracy of the polymerase chain reaction tests was evolving; some patients required multiple tests and were clinically treated as having COVID-19. Furthermore, with regard to our findings regarding who did not return after receiving antibiotics, some of these patients may have been treated for bacterial etiologies, which may explain their low return rate.

One of our assumptions in our regression analysis was that patients who did not have lab tests ordered, and thus had no reported values, were classified as normal. This assumption was made because clinicians did not deem the tests to be relevant to the patient’s diagnosis or disposition. As these tests were not available upon discharge, it did not contribute to the overall decision-making process regarding the patient’s disposition. Patients in these instances were presenting overall well-appearing, and during a time of limited resources, ordering such tests was unnecessary. In addition, our analytical testing indicated that separating those patients who did not have labs ordered into their own category resulted in unstable modeling estimates, therefore necessitating the combining of categories. Because of this, there is the potential that some patients were misclassified. However, we believe this proportion to be low and to have not influenced our estimates in any meaningful way.

## CONCLUSION

During the initial surge of the pandemic in the New York City metropolitan area, there was limited knowledge of COVID-19 and its clinical course in patients who presented to the ED. Despite this lack of knowledge, our 72-hour return rate was relatively low, even with an extremely high rate of patients who were presenting with COVID-19. As COVID-19 diagnostic tools and treatment algorithms evolve, we need to better understand the factors that may contribute to a patient returning to the ED. As many patients with COVID-19 can be discharged at the initial time of evaluation, programs and follow-up procedures tailored to these patients should be implemented and investigated.

## Supplementary Information





## Figures and Tables

**Table 1:** Inclusion criteria met by study sample (N=18,599).

	No. (%)
Inclusion criteria description chief complaint relating to viral illness	
Flu	4,528 (24.35%)
Fever	4,372 (23.51%)
Cough	3,870 (20.81%)
Shortness of breath	3,223 (17.33%)
Chest pain	741 (3.98%)
COVID-19	469 (2.52%)
Cold	226 (1.22%)
Upper respiratory infection	46 (0.25%)
Category of discharge diagnosis based on ICD-10-CM codes designated by clinician	
Signs and symptoms involving the respiratory system	4,317 (23.21%)
General symptoms and signs	3,353 (18.03%)
Infectious and communicable diseases	2,873 (15.45%)
Flu-like symptoms including fever, malaise, fatigue, dizziness	2,337 (12.57%)
No ICD-10 code available	2,275 (12.23%)
Respiratory infection	2,127 (11.44%)
COVID-19	331 (1.78%)
Pain	282 (1.52%)
Signs and symptoms involving the gastrointestinal system	233 (1.25%)
Encounter for other disease, disorder, or symptom	154 (0.83%)
Headache	77 (0.41%)
Signs and symptoms involving the circulatory system	54 (0.29%)
Other symptoms and signs involving the circulatory and respiratory system	43 (0.23%)
Syncope and collapse	41 (0.22%)
Signs and symptoms of mental and behavioral disorders	22 (0.12%)
Abnormal findings through testing and on examination	20 (0.11%)
Signs and symptoms involving the musculoskeletal system	20 (0.11%)
Injuries and environmental health hazards	16 (0.09%)
Disturbances of smell and taste	12 (0.06%)
Encounter for circumstances and disorders related to maternal care, pregnancy, and reproduction	10 (0.05%)
Complications of surgical and medical care, not elsewhere classified	2

*COVID-19*, coronavirus disease 2019; *ICD-10-CM*, International Classification of Diseases, 10th Revision, Clinical Modification.

**Table 2:** Demographic characteristics of patients with suspected COVID-19 (N=18,599).

Demographic information	No. (%)
Age, median (IQR), [Range]	46, (34–58), [18–104]
Gender	
Female	9,437 (50.74%)
Male	9,162 (49.26%)
Race	
Black	3,287 (17.67%)
Asian	1,499 (8.06%)
White	6,365 (34.22%)
Native American/Alaskan/Hawaiian/Pacific Islander/unknown	1,222 (6.57%)
Other/multiracial	6,226 (33.47%)
Ethnicity	
Hispanic or Latinx	5,384 (28.95%)
Not Hispanic or Latinx	11,677 (62.78%)
Unknown	1,538 (8.27%)
Prior hospitalization within past 6 months	
0	17,304 (93.04%)
1–2	1,137 (6.11%)
≥3	158 (0.58%)
Insurance	
Commercial	13,983 (75.18%)
Medicaid	869 (4.67%)
Medicare	1,322 (7.11%)
Other/unknown	114 (0.61%)
Self-pay/uninsured	2,311 (12.43%)
ESI Triage Level	
1	16 (0.09%)
2	2,386 (12.83%)
3	9,722 (52.27%)
4	6,312 (33.94%)
5	163 (0.88%)
Language	
English	14,694 (79.00%)
Spanish	2,871 (15.44%)
Other	576 (3.10%)
Unknown	458 (2.46%)
Medical history	
Cancer	573 (3.08%)
Chronic pulmonary disease	21 (0.11%)
Cardiovascular disease	1,130 (6.08%)
COVID-19	15 (0.08%)
CVA/TIA	179 (0.96%)
Diabetes	1,702 (9.15%)
Gastrointestinal disorder	150 (0.81%)
Hematologic	22 (0.12%)
Hypertension	3,209 (17.25%)
Immunologic disease	115 (0.62%)
Obesity	378 (2.03%)
Pulmonary disease	1,715 (9.22%)
Renal disease	138 (0.74%)
Smoking	36 (0.19%)
Transplant	10 (0.05%)
Venous thrombotic disease	193 (1.04%)
BMI Class	
Missing/unknown	8,436 (45.36%)
Underweight	123 (0.66%)
Normal weight	2,596 (13.96%)
Pre-obesity	3,845 (20.67%)
Obesity class I	2,213 (11.90%)
Obesity class II	864 (4.65%)
Obesity class III	522 (2.81%

*IQR*, interquartile range; *ESI*, Emergency Severity Index; *COVID-19*, coronavirus disease 2019.

*CVA*, cerebral vascular accident; *TIA*, transient ischemic attack; *BMI*, body mass index.

**Table 3:** Odds ratio estimates for multivariate analysis: 72-hour return.

Effect	Point estimate	95% Wald confidence limits	
Age (Estimate × [n] year)	1.03	1.03	1.04
Age (Estimate × 10 [n] year)	1.37	1.28	1.47
Gender			
Female	1.00	(Reference)	
Male	1.41	1.17	1.70
Insurance status			
Employee/managed care	1.00	(Reference)	
Medicaid	1.23	0.82	1.83
Medicare	0.66	0.49	0.90
Other/unknown	1.89	0.74	4.83
Self-pay/uninsured	0.64	0.45	0.92
Initial chief complaint			
Fever			
Not present	1.00	(Reference)	
Present	1.78	1.44	2.20
Flu			
Not present	1.00	(Reference)	
Present	1.56	1.20	2.01
Past medical history			
Diabetes			
Not present	1.00	(Reference)	
Present	1.33	1.04	1.69
Renal			
Not present	1.00	(Reference)	
Present	2.07	1.14	3.76
Number of hospitalizations within past 6 months			
0	1.00	(Reference)	
1–2	1.63	1.21	2.18
≥3	1.51	0.80	2.88
Triage ESI Level			
1	5.34	1.07	26.55
2	1.29	1.03	1.62
3	1.00	(Reference)	
4	0.69	0.51	0.92
5	0.52	0.07	3.77
Vital signs from triage to discharge			
Temperature			
Normal to normal	1.00	(Reference)	
Abnormal to abnormal	2.43	1.83	3.218
Abnormal to normal	1.65	1.26	2.16
Normal to abnormal	1.78	1.06	2.98
Systolic BP			
Normal to normal	1.00	(Reference)	
Abnormal to abnormal	0.68	0.53	0.86
Abnormal to normal	0.68	0.51	0.92
Normal to abnormal	0.72	0.43	1.18
Respiratory rate			
Normal to normal	1.00	(Reference)	
Abnormal to abnormal	2.17	1.57	3.00
Abnormal to normal	1.57	1.15	2.14
Normal to abnormal	1.17	0.64	2.13
Radiology findings of chest radiograph			
Normal	1.00	(Reference)	
Abnormal	2.54	2.00	3.22
Not done	0.93	0.70	1.22
Therapies administered			
Antibiotics in the ED			
Not administered	1.00	(Reference)	
Administered	1.52	1.11	2.07
Discharged on antibiotics			
Not discharged on antibiotics	1.00	(Reference)	
Discharged on antibiotics	0.12	0.05	0.30
Discharged on corticosteroids			
Not Discharged on corticosteroids	1.00	(Reference)	
Discharged on corticosteroids	0.12	0.02	0.89
Laboratory values			
AST			
Normal/missing	1.00	(Reference)	
High	1.63	1.30	2.07
Bicarbonate			
Normal/missing	1.00	(Reference)	
High	4.66	1.84	11.79
Low	1.98	0.62	6.35
Neutrophils			
Normal	1.00	(Reference)	
Low	0.31	0.08	1.31
High	1.32	1.03	1.69
Platelets			
Normal	1.00	(Reference)	
Low	1.68	1.19	2.38
High	0.64	0.23	1.80
Disposition on first visit			
Discharge	1.00	(Reference)	
Against medical advice	2.97	1.29	6.84

The area under the receiver operating characteristic curve for the regression model was 0.84.

ESI, Emergency Severity Index; BP, blood pressure.

*ED*, emergency department; *AST*, aspartate aminotransferase; *CRP*, C-reactive protein.
