# Supplementary material for: Characteristics of Suspected COVID-19 Discharged Emergency Department Patients Who Returned During the First Wave
Source: West J Emerg Med. 2023 Apr 3;24(3):405–15. doi: 10.5811/westjem.58717 (PMC10284524; doi:10.5811/westjem.58717)
Supplement: Supplementary file 1 [file wjem-24-405-s001.docx]

**Appendix A.** Terms used in a string search of ED discharge instructions to determine if patients were suspected to have COVID-19.

| **Terms Determining Suspicion of COVID-19 Diagnosis** | **Terms to Exclude** |
| --- | --- |
| COVID | Abdominal pain |
| 14 Day | Stomach pain |
| Quarantine | Stomach flu |
| Coronavirus | Urinary - Urinary tract infection |
| Viral | UTI |
| Upper respiratory infection | Chest pain |
| URI |  |
| Cough |  |
| PNA |  |
| Pneumonia |  |
| Pulse oximeter |  |
| Oxygen reading device |  |
| Viral resp |  |
| Upper resp |  |
| Flu |  |
| Isolate |  |
| Stay home |  |
| Stay at home |  |
