# Supplementary material for: Characteristics of Suspected COVID-19 Discharged Emergency Department Patients Who Returned During the First Wave
Source: West J Emerg Med. 2023 Apr 3;24(3):405–15. doi: 10.5811/westjem.58717 (PMC10284524; doi:10.5811/westjem.58717)
Supplement: Supplementary file 2 [file wjem-24-405-s002.docx]

**Appendix B.** Vital Sign and Laboratory Reference Ranges.

| **Vitals Results** |  | **Normal** | **Unit of Measure** |
| --- | --- | --- | --- |
| Temperature | | 96.8-100.4 | °F |
| Systolic BP | | 90 - 140 | mm Hg |
| Heart Rate | | 60-100 | bpm |
| Respiration Rate | | 12-20 | breaths/min |
| Initial SpO2 | | ≥ 92 | % |
|  | | | |
| **Laboratory Results** | **Abnormal** | **Normal** | **Units of Measure** |
| White blood cell count | Low ≤ 3.79, High ≥ 10.81 | 3.80 – 10.80 | K/µL |
| Lymphocytes, ABS | Low ≤ 12.9, High ≥ 43.1 | 13 – 43.0 | K/µL |
| Neutrophils | Low ≤ 42.1, High ≥ 77.1 | 42.2 – 77.0 | % |
| Hemoglobin | Low ≤ 11.4, High ≥18.1 | 11.5 – 18.0 | g/dL |
| Platelet count | Low ≤ 129, High ≥441 | 130 - 440 | K/µL |
| Sodium | Low ≤ 131, High ≥ 147 | 132 – 146 | mmol/L |
| Potassium | Low ≤ 3.4, High ≥ 5.4 | 3.5 – 5.3 | mmol/L |
| Chloride | Low ≤ 95, High ≥111 | 96-110 | mmol/L |
| Serum carbon dioxide | Low ≤ 16, High ≥ 33 | 16 - 32 | mmol/L |
| Blood Urea Nitrogen | High ≥ 24 | 0 - 23 | mg/dL |
| Serum Creatinine | High ≥ 1.31 | 0 – 1.30 | mg/dL |
| Glucose | Low ≤ 69, High ≥ 181 | 70 - 180 | mg/dL |
| Alanine aminotransferase | > 79 | < 5-78 | U/L |
| Aspartate aminotransferase | > 42 | < 5 - 41 | U/L |
| Total bilirubin serum | ≥ 2.1 | ≤2.0 | mg/dL |
| D-dimer assay | High >905 | < 904 | ng/mL |
| Ferritin, serum | High ≥ 400 | ≤ 399 | ng/mL |
| Troponin I | > 0.055 | ≤ 0.054 | ng/L |
| Troponin T, high sensitivity | ≥ 28 | <6 - 27 | ng/L |

Abbreviations: BP, Blood Pressure; SpO2, Peripheral Oxygen Saturation; ABS, Absolute
